# Loss of LSR affects epithelial barrier integrity and tumor xenograft growth of CaCo-2 cells

**DOI:** 10.18632/oncotarget.10425

**Published:** 2016-07-06

**Authors:** Bernd A. Czulkies, Justin Mastroianni, Lisa Lutz, Sarah Lang, Carsten Schwan, Gudula Schmidt, Silke Lassmann, Robert Zeiser, Klaus Aktories, Panagiotis Papatheodorou

**Affiliations:** ^1^ Institute of Experimental and Clinical Pharmacology and Toxicology, Albert-Ludwigs-University (ALU), Freiburg, Germany; ^2^ Department of Hematology and Oncology, University Medical Center, ALU, Freiburg, Germany; ^3^ Department of Pathology, University Medical Center, ALU, Freiburg, Germany; ^4^ German Consortium for Translational Cancer Research (DKTK) and German Cancer Research Center (DKFZ), Heidelberg, Germany; ^5^ Centre for Biological Signalling Studies (BIOSS), ALU, Freiburg, Germany; ^6^ Freiburg Institute for Advanced Studies (FRIAS), ALU, Freiburg, Germany; ^7^ Present address: Institute of Pharmaceutical Biotechnology. University of Ulm, Ulm, Germany; ^8^ Present address: Institute of Pharmacology and Toxicology, University of Ulm Medical Center, Ulm, Germany

**Keywords:** tumor growth, xenograft, epithelial barrier, cell morphology, cell-cell contact

## Abstract

The lipolysis-stimulated lipoprotein receptor (LSR) is a lipoprotein receptor, serves as host receptor for clostridial iota-like toxins and is involved in the formation of tricellular contacts. Of particular interest is the role of LSR in progression of various cancers. Here we aimed to study the tumor growth of LSR-deficient colon carcinoma-derived cell lines HCT116 and CaCo-2 in a mouse xenograft model. Whereas knockout of LSR had no effect on tumor growth of HCT116 cells, we observed that CaCo-2 LSR knockout tumors grew to a smaller size than their wild-type counterparts. Histological analysis revealed increased apoptotic and necrotic cell death in a tumor originating from LSR-deficient CaCo-2 cells. LSR-deficient CaCo-2 cells exhibited increased cell proliferation *in vitro* and an altered epithelial morphology with impaired targeting of tricellulin to tricellular contacts. In addition, loss of LSR reduced the transepithelial electrical resistance of CaCo-2 cell monolayers and increased permeability for small molecules. Moreover, LSR-deficient CaCo-2 cells formed larger cysts in 3D culture than their wild-type counterparts. Our study provides evidence that LSR affects epithelial morphology and barrier formation in CaCo-2 cells and examines for the first time the effects of LSR deficiency on the tumor growth properties of colon carcinoma-derived cell lines.

## INTRODUCTION

The lipolysis-stimulated lipoprotein receptor (LSR; also denoted as Lisch7 or angulin-1) is a type I single-pass transmembrane protein that is mainly expressed in the liver but also in the intestine and in many other tissues [1]. As the name already suggests, LSR was firstly described as a lipoprotein receptor in the liver that contributes to the clearance of chylomicron remnants from the blood [1–4]. Later it turned out that LSR fulfills additional functions in mammalian cells. For instance, LSR is involved in the formation of tricellular tight junctions in the mammary epithelial cell line EpH4 [5, 6]. In addition, our laboratory showed that LSR acts as the cell entry receptor for clostridial iota-like toxins, namely CDT from *Clostridium difficile, Clostridium perfringens* iota-toxin and *Clostridium spiroforme* toxin [7, 8]. More recent data indicate that LSR is critical for proper blood-brain barrier (BBB) formation and function during development at embryonic day 14.5 [9]. Noteworthy, mice with a homozygous deletion of LSR are nonviable and die between embryonic days 12.5 and 15.5 [1].

An increasing number of studies suggests that LSR might be implicated in the progression of various cancers. For instance, LSR is one of the most up-regulated genes related to development of visible metastasis in a mouse mammary tumor model [10]. Furthermore, LSR promotes invasion and cellular movement in bladder cancer and aggressive breast cancer behavior [11, 12]. LSR expression levels in human colon cancer are associated with a poor prognosis [13]. Very recently, Shimada and colleagues reported that knockdown of LSR induced cell migration, invasion and proliferation in endometrial cancer cells [14]. Here, we aimed to study in a mouse xenograft model the consequences of LSR knockout on tumor growth of two colon-derived cancer cell lines (CaCo-2 and HCT116). We recently used a CRISPR/Cas9-based approach to knockout LSR in the human colorectal carcinoma cell line HCT116 [15]. Here we generated with the same approach an LSR knockout in the human colorectal adenocarcinoma cell line CaCo-2. Our study examines for the first time the effects of LSR deficiency on the tumor growth properties of colon carcinoma-derived cell lines. We also used CaCo-2 cells to study the role of LSR in formation of cell-cell contacts in epithelial cell monolayers, in growth of cysts in a 3D cell culture model, and in establishment of epithelial barrier function. We found that LSR is required for maintenance of epithelial barrier integrity and for tumor xenograft growth of CaCo-2 cells.

## RESULTS

### Generation and evaluation of a CaCo-2 LSR knockout cell line

**Figure 1:**
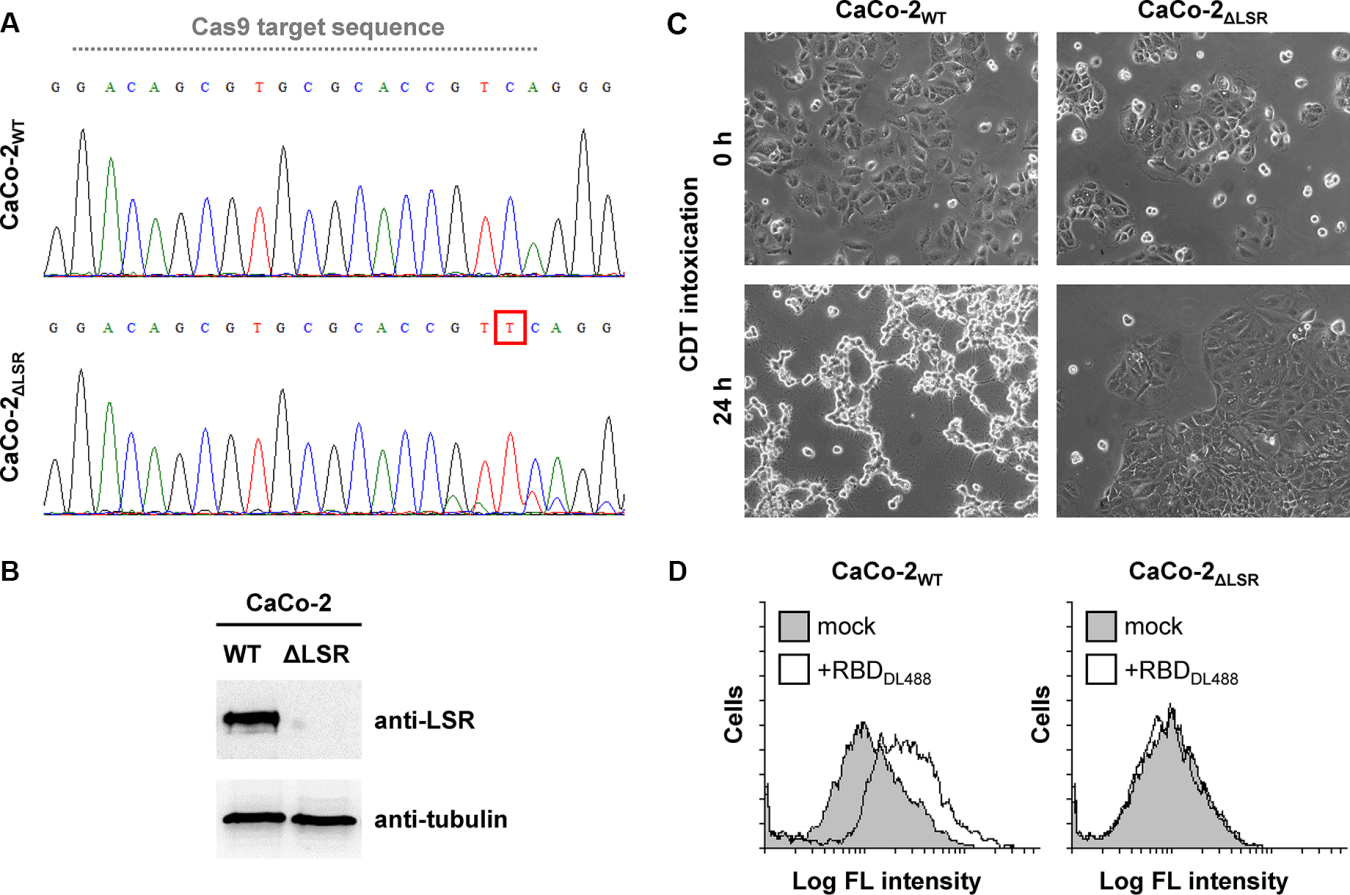
Evaluation of the CRISPR/Cas9-generated CaCo-2 LSR knockout cells (**A**) DNA sequencing chromatograms for CaCo-2_WT_ and CaCo-2_ΔLSR_ cells covering a sequence within exon 2 of the LSR gene. Grey dotted line indicates the Cas9 target sequence. A red box highlights the base pair insertion (thymine) in exon 2 of the LSR gene from CaCo-2_ΔLSR_ cells. (**B**) LSR immunoblot with whole-cell lysates from CaCo-2_WT_ (WT) and CaCo-2_ΔLSR_ (ΔLSR) cells (top panel). Equal loading of samples was verified by detecting tubulin with a specific antibody (bottom panel). (**C**) Intoxication of CaCo-2_WT_ and CaCo-2_ΔLSR_ cells with CDT. Microscopic images were taken at indicated time points after toxin addition. (**D**) Suspensions of CaCo-2_WT_ and CaCo-2_ΔLSR_ cells were incubated with the receptor-binding domain of CDT labeled with DyLight488 (RBD_DL488_) or were left untreated (mock) prior to FACS analysis. FACS results are presented as histogram plots and single cell events (cells) are plotted against the intensity of cell-surface bound fluorescence (Log FL intensity). White peaks indicate the fluorescence distribution of cells incubated with RBD_DL488_ and grey peaks represent mock-treated cells with no addition of fluorescence-labeled protein.

LSR-deficient HCT116 cells were already available in our laboratory [15]. However, since the human colorectal adenocarcinoma cell line CaCo-2 is an important cancer cell model, we aimed to disrupt the LSR gene also in CaCo-2 cells via CRISPR/Cas9. We were able to isolate a CaCo-2 clone with a base pair insertion between positions 518/519 of the LSR coding region (termed CaCo-2_ΔLSR_ in this study). The base pair insertion leads to a frame shift mutation in exon 2 (Figure 1A), yielding a non-functional protein that consists only of a short segment of the extracellular portion of LSR. Immunoblot analysis with an LSR-specific antibody confirmed the absence of LSR expression in CaCo-2_ΔLSR_ cells (Figure 1B). To further confirm the lack of LSR expression in CaCo-2_ΔLSR_ cells, we intoxicated wild-type and LSR-deficient CaCo-2 cells with the *C. difficile* binary toxin CDT, which requires LSR for host cell binding and entry [7]. As expected, CDT-induced cell rounding was only observed in wild-type CaCo-2 cells (CaCo-2_WT_), whereas CaCo-2_ΔLSR_ cells remained resistant towards CDT (Figure 1C). We then tested binding of the receptor-binding domain (RBD) of CDT to the surface of wild-type and LSR-deficient CaCo-2 cells via flow cytometry. For this purpose, the RBD of CDT was labeled with a green fluorescent dye (DyLight488). Binding of DyLight488-labeled RBD (RBD_DL488_) to the cell surface, indicated by the increase of green fluorescence of the RBD_DL488_-bound cell population, was only observed in CaCo-2_WT_ but not in CaCo-2_ΔLSR_ cells (Figure 1D). Taken together, these results confirmed that the LSR gene is disrupted in CaCo-2_ΔLSR_ cells.

### LSR is required for efficient CaCo-2 tumor growth in a mouse xenograft model

Several studies have shown that LSR might be implicated in the progression of various cancers [10–13]. These observations prompted us to study the role of LSR in tumor growth and behavior of CaCo-2 and HCT116 wild-type and LSR-deficient cells in a mouse xenograft model.

**Figure 2:**
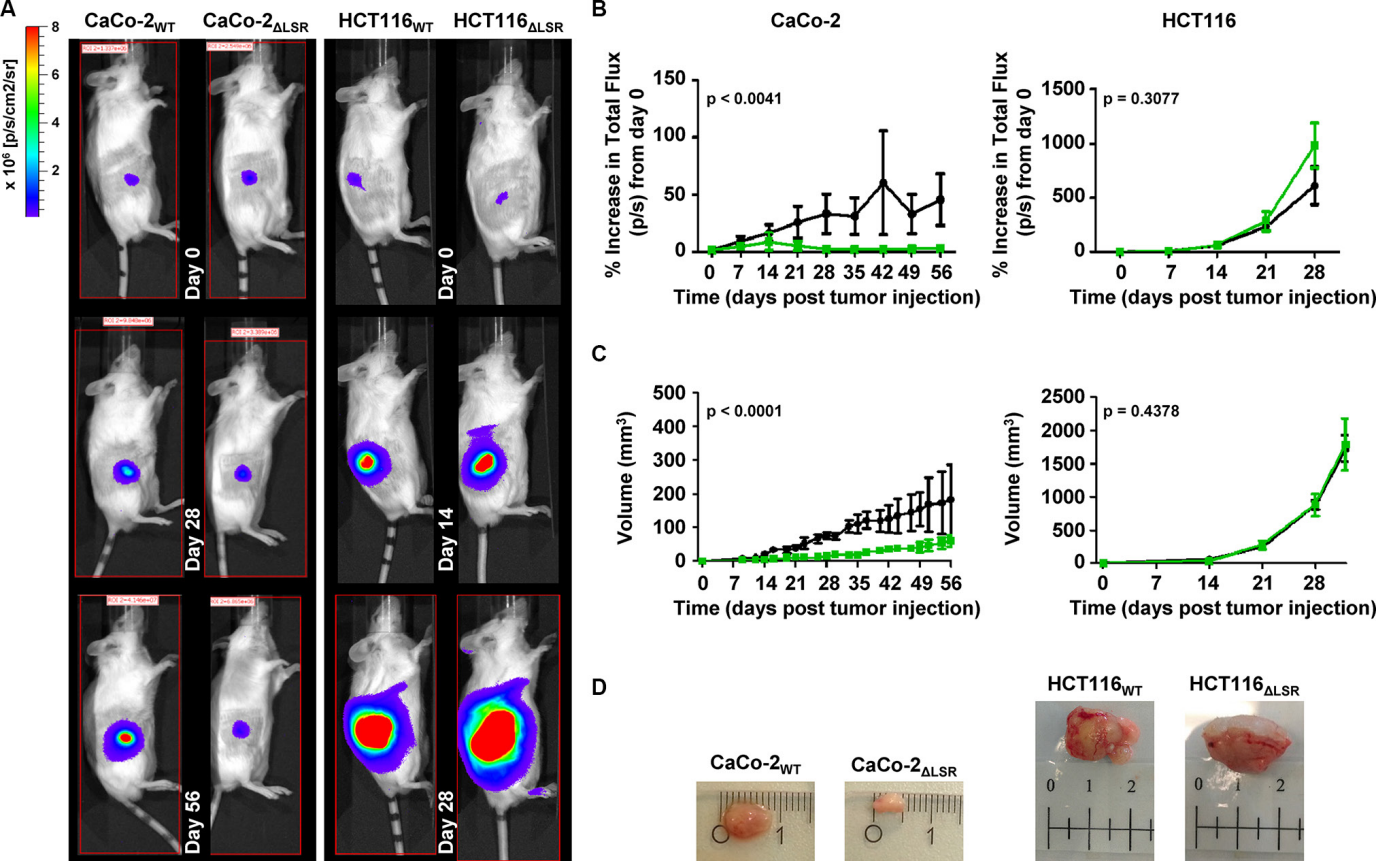
Growth characteristics of wild-type and LSR-deficient CaCo-2 and HCT116 tumors in immunodeficient mice Sixteen Rag2 KO mice were divided into four groups and 2 × 10^6^ luciferase-expressing CaCo-2_WT_ (group I) and CaCo-2_ΔLSR_ cells (group II) or 5 × 10^5^ luciferase-expressing HCT116_WT_ (group III) and HCT116_ΔLSR_ cells (group IV) were subcutaneously injected into their right flank. One mouse from group II died 21 days after injection of cells. (**A**) *In vivo* bioluminescence imaging of the growing tumors in representative mice at day 0, 28 and 56 (CaCo-2_WT_ or CaCo-2_ΔLSR_; left panel) and day 0, 14 and 28 (HCT116_WT_ or HCT116_ΔLSR_; right panel). Bioluminescence intensity is shown in colors from blue (low) to red (high). (**B**) Quantification of tumor growth based on its bioluminescence. The increase of the average total flux from tumors of each mice group (Rag2 KO + wild-type CaCo-2 or HCT116 (black line) and Rag2 KO + LSR-deficient CaCo-2 or HCT116 (green line)) is shown as a function of time. Error bars represent ± SEM. (**C**) The average tumor volume (in mm^3^) of each mice group (Rag2 KO + wild-type CaCo-2 or HCT116 (black line) and Rag2 KO + LSR-deficient CaCo-2 or HCT116 (green line)) is shown as a function of time. Error bars represent ± SEM. (**D**) A representative tumor from each mice group is shown. Ruler shows cm.

To this end, an equal number of luciferase-expressing HCT116_WT_ or HCT116_ΔLSR_ cells and CaCo-2_WT_ or CaCo-2_ΔLSR_ cells, respectively, was subcutaneously injected into the right flank of immunodeficient mice and tumor growth was monitored for a period of 28 (HCT116 cells) or 56 (CaCo-2 cells) days. Bioluminescence imaging (BLI) (Figure 2A and 2B) and volumetric measurements (Figure 2C and 2D) of the tumors revealed that wild-type HCT116 cells developed much larger tumors at day 28 than wild-type CaCo-2 cells at day 56. However, of major importance was the observation that CaCo-2_ΔLSR_ cells developed much smaller tumors in mice than their respective wild-type counterparts (Figure 2A–2D, left panel). No significant differences in tumor size were observed between tumors originating from wild-type and LSR-deficient HCT116 cells (Figure 2A–2D, right panel).

**Figure 3:**
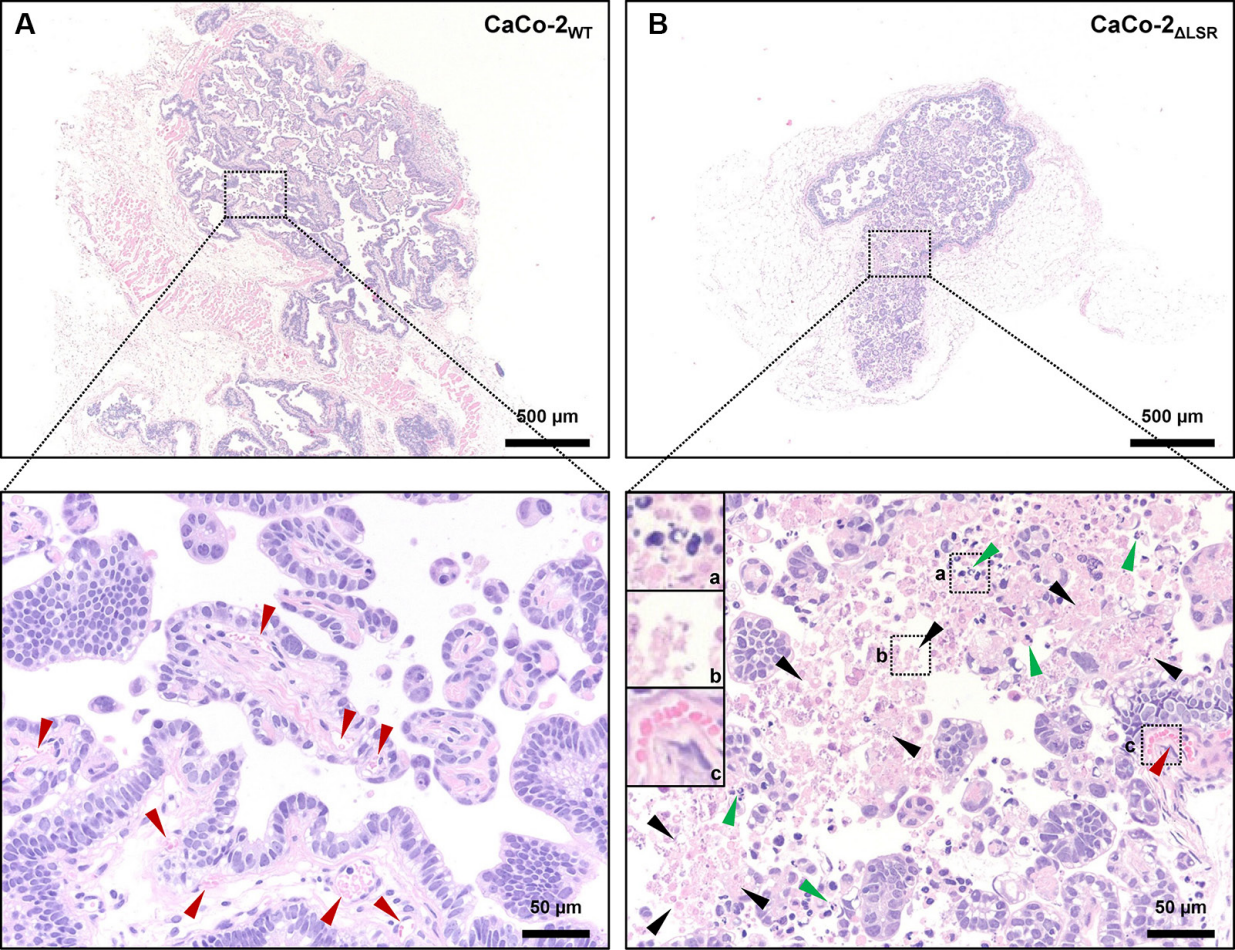
Histology of CaCo-2_WT_ and CaCo-2_ΔLSR_ xenograft tumors The panels show Hematoxylin and Eosin (H&E) stainings of representative sections of (**A**) CaCo-2_WT_ and (**B**) CaCo-2_ΔLSR_ tumors. Upper panels illustrate the difference of the xenograft tumor size (larger tumor in (A)). Lower panels show enlargements from upper panels (dotted boxes), with marked blood vessels in an accompanying stromal component (red arrows) in CaCo-2_WT_ (left) and marked apoptosis with karyorrhexis and intact plasma membrane (green arrows) as well as necrosis with karyorrhexis, karyolysis and disrupted plasma membrane (black arrows) in CaCo-2_ΔLSR_ (right) xenograft tumors.

Hence, we next focused on the histological analysis of tissue specimens from the CaCo-2_WT_ and CaCo-2_ΔLSR_ tumors. Hematoxylin and eosin (H&E) staining of histological sections of tissue specimens from the CaCo-2_WT_ and CaCo-2_ΔLSR_ tumors revealed a papillary growth pattern for both tumors. However, the CaCo-2_ΔLSR_ tumor displayed a less prominent stromal component than the CaCo-2_WT_ tumor, with clear stromal reaction, including blood vessels. Moreover, massive apoptotic and necrotic cell death occurred in the CaCo-2_ΔLSR_ tumor (Figure 3A and 3B).

### LSR-deficient CaCo-2 cells exhibit increased cell proliferation *in vitro*

**Figure 4:**
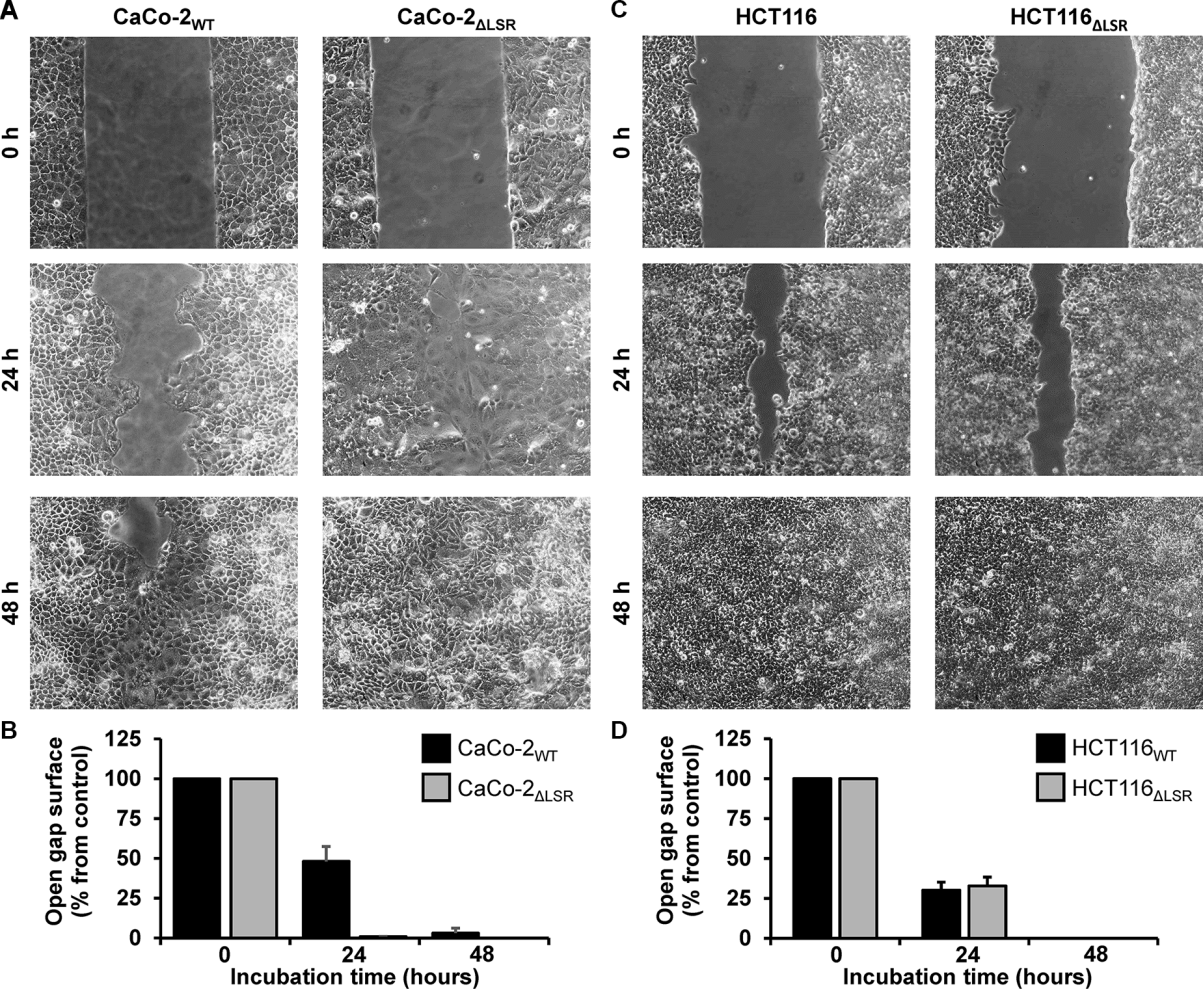
Assessing the proliferative potential of wild-type and LSR-deficient CaCo-2 and HCT116 cells with a cell exclusion zone assay (**A**) Gap closure of CaCo-2_WT_ and CaCo-2_ΔLSR_ cells was monitored microscopically 0, 24 and 48 h after generating a cell-free gap by removing a silicon-based culture insert from the confluently growing monolayer. (**B**) Quantification of the cell exclusion zone assay shown in (A). The open gap surface was measured and results are shown relative to the initial open gap surface, which was set to 100%. Bar graphs represent quantitative analysis of three independent experiments. The values represent mean ± SEM. (**C**, **D**) As shown in (A, B) but with HCT116_WT_ and HCT116_ΔLSR_ cells.

**Figure 5:**
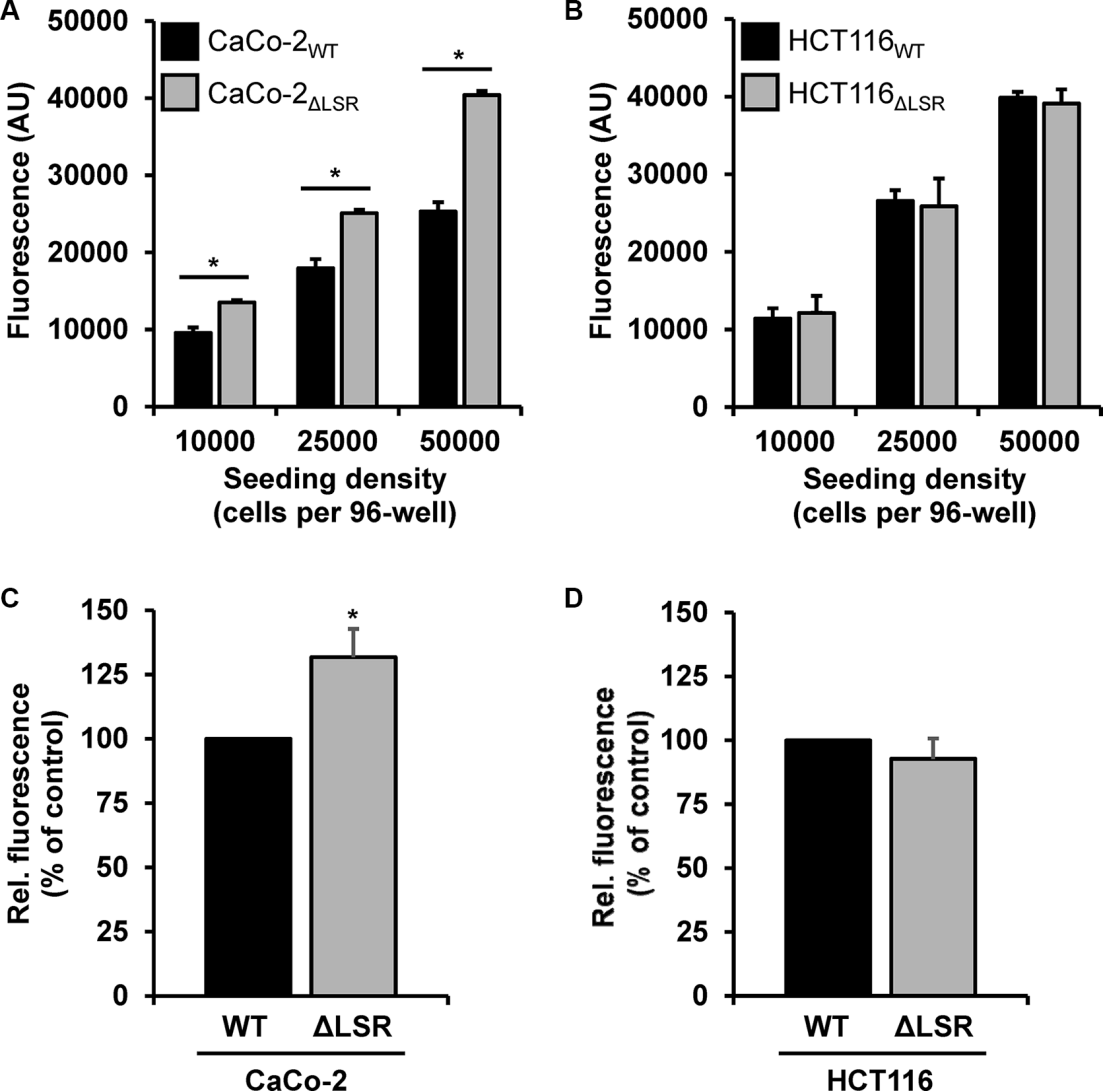
Assessing the proliferative potential of wild-type and LSR-deficient CaCo-2 and HCT116 cells with a cell viability and an EdU proliferation assay (**A**, **B**) Cell viability assay. Absolute resorufin fluorescence values are presented in arbitrary units (AU). Black bars represent fluorescence values obtained with (A) CaCo-2_WT_ or (B) HCT116_WT_ cells and grey bars with (A) CaCo-2_ΔLSR_ or (B) HCT116_ΔLSR_ cells, respectively. Bar graphs represent quantitative analysis of three independent experiments. The values represent mean ± SEM; *p < 0.05. (C, D) EdU proliferation assay. Fluorescent EdU incorporation into (**C**) CaCo-2_ΔLSR_ or (**D**) HCT116_ΔLSR_ cells (ΔLSR; grey bars) is presented relative to the fluorescence values obtained with their wild-type counterparts (WT, black bars), which were set to 100%. Bar graphs represent quantitative analysis of three independent experiments. The values represent mean ± SEM; *p < 0.05.

Next, we asked if the reduced size of the CaCo-2 LSR knockout tumor is due to a decreased cell proliferation of LSR-deficient CaCo-2 cells. To test this hypothesis, we performed a cell exclusion zone assay. For this purpose, CaCo-2_WT_ and CaCo-2_ΔLSR_ cells, as well as HCT116_WT_ and HCT116_ΔLSR_ cells, were grown to confluency in a silicon-based culture insert, allowing cells to grow only in the designated areas. A cell-free gap of approximately 500 μm was created after removing the culture insert and closure of the gap by proliferating and/or migrating cells could be monitored microscopically. For CaCo-2_WT_ cells, it took about 48 h to close the gap completely, whereas for CaCo-2_ΔLSR_ the gap closure already occurred after 24 h (Figure 4A and 4B). No difference was observed in gap closure speed between HCT116_WT_ and HCT116_ΔLSR_ cells (Figure 4C and 4D). In addition, a cell viability assay based on the ability of living cells to convert the redox dye resazurin into its fluorescent end product resorufin was performed [16]. As shown in Figure 5A, CaCo-2_ΔLSR_ cells exhibited a significantly higher cell viability than CaCo-2_WT_ cells at three different initial seeding densities. In comparison, no difference in cell viability was observed for HCT116_WT_ and HCT116_ΔLSR_ cells (Figure 5B). To verify these findings, a fluorescence-based replication assay based on EdU (5-ethynyl-2′-deoxyuridine) incorporation into replicating DNA was performed. With this approach, we observed that CaCo-2_ΔLSR_ cells incorporated significantly more EdU than CaCo-2_WT_ cells (Figure 5C). As expected, also in this assay HCT116_WT_ and HCT116_ΔLSR_ cells showed no difference in EdU incorporation (Figure 5D). Taken together, these results indicate that the loss of LSR leads to an increase of the replicative potential of CaCo-2 cells. The reduced size of the CaCo-2 LSR knockout tumor in the mouse xenograft model is not likely to be explained by reduced cell proliferation.

### Loss of LSR leads to an aberrant epithelial sheet geometry and mislocalization of tricellulin in CaCo-2 cells

**Figure 6:**
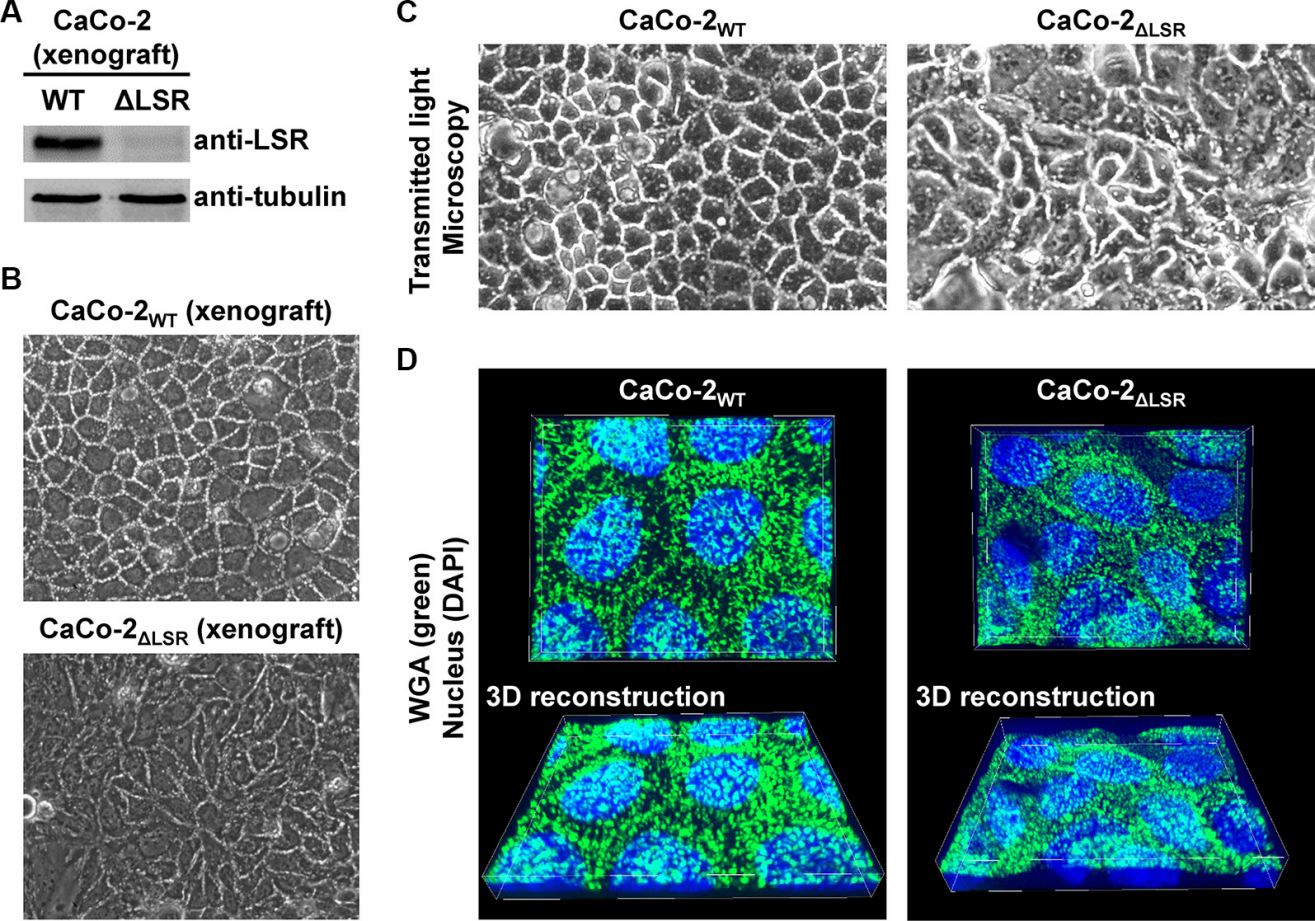
Morphology of cell monolayers from CaCo-2_WT_ versus CaCo-2_ΔLSR_ cells (**A**) CaCo-2_WT_ and CaCo-2_ΔLSR_ cells were isolated from tumor tissue and plated into cell culture dishes for confluent growth, followed by immunodetection of LSR in whole-cell lysates (**B**, **C**) Transmitted light microscopy images of confluent cell monolayers from CaCo-2_WT_ and CaCo-2_ΔLSR_ cells isolated from a xenograft tumor (B) or from cells that grew only in cell culture (C). (**D**) The cell-surface of confluently growing CaCo-2_WT_ and CaCo-2_ΔLSR_ cells was stained with wheat-germ agglutinin (WGA; green) and visualized by confocal fluorescence microscopy (top panel). Cell nuclei were stained with DAPI (blue). 3D reconstructions (bottom panel) to obtain a tilted view on the cell monolayer were generated with MetaMorph image analysis software.

The increased proliferative potential of LSR-deficient CaCo-2 cells *in vitro* indicated that growth control mechanisms are impaired in these cells. However, contact inhibition of cells is important for the acquisition of an epithelial cell morphology (Abercrombie, 1979). To test whether CaCo-2 LSR knockout cells still maintain an epithelial-like growth phenotype, we dissociated cells from CaCo-2_WT_ or CaCo-2_ΔLSR_ tumor tissue and recultivated them in petri dishes for microscopic analysis. LSR-deficiency of the xenograft-derived CaCo-2_ΔLSR_ cells was confirmed via immunoblotting with an LSR-specific antibody (Figure 6A). We observed that the xenograft-derived CaCo-2_ΔLSR_ cells exhibited an aberrant epithelial sheet geometry after re-cultivation and formation of confluent monolayers in petri dishes when compared to re-cultivated cells from a CaCo-2_WT_ tumor (Figure 6B). The same disordered phenotype was also observed for the initially established CaCo-2_ΔLSR_ cell line upon growth to confluency (Figure 6C), indicating that these morphological changes were not induced by xenograft growth in mice. These observations were supported by confocal fluorescence images of confluent epithelial monolayers of CaCo-2_WT_ and CaCo-2_ΔLSR_ cells stained with fluorescent wheat germ agglutinin (Figure 6D).

**Figure 7:**
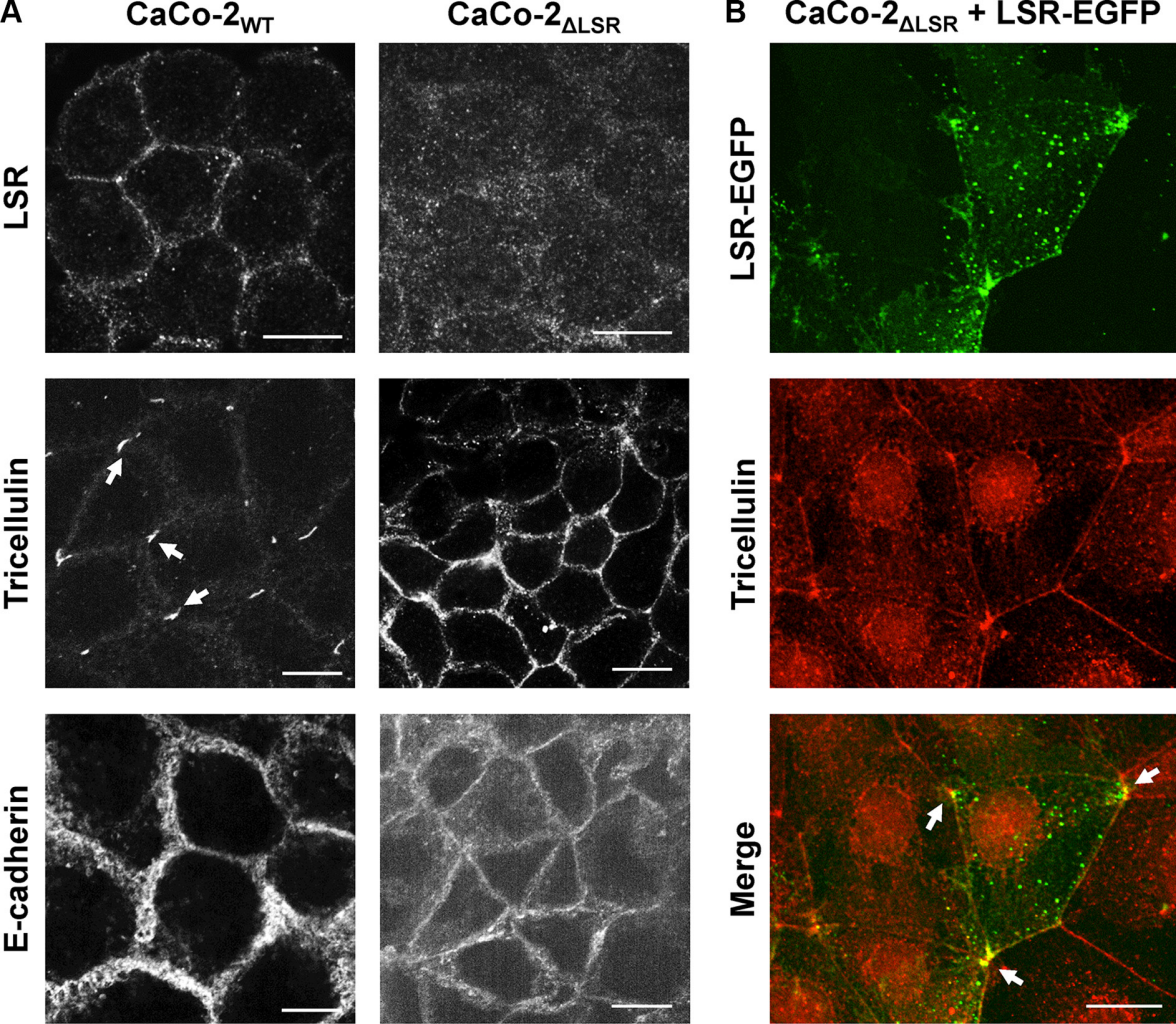
Staining of bi- and tricellular marker proteins in cell monolayer of wild-type and LSR-deficient CaCo-2 cells (**A**) Immunostaining of LSR (top panel), tricellulin (middle panel) and E-cadherin (bottom panel) in CaCo-2_WT_ and CaCo-2_ΔLSR_ cells with specific antibodies and analysis of the intracellular distribution of the respective proteins via confocal fluorescence microscopy. (**B**) Reconstitution of LSR expression in CaCo-2_ΔLSR_ cells. CaCo-2_ΔLSR_ cells were transfected with a plasmid that leads to ectopic expression of LSR-EGFP (green signal). Cells were immunostained for tricellulin (red signal). Arrows indicate accumulation of LSR-EGFP and tricellulin in tricellular contacts. Scale bars represent 10 μM.

To test whether bi- or tricellular cell-cell contacts are affected by the loss of LSR, we performed immunostaining of LSR, tricellulin and E-cadherin in confluent monolayers of CaCo-2_WT_ and CaCo-2_ΔLSR_ cells. Tricellulin is a protein that is found predominantly at the sites in which three epithelial cells meet (tricellular junctions) [17]. E-cadherin is found in adhesion junctions and initiates intercellular contacts [18]. Fluorescence images confirmed the lack of LSR expression in CaCo-2_ΔLSR_ cells (Figure 7A, upper panel). Interestingly, in CaCo-2_ΔLSR_ cells tricellulin did not localize exclusively at tricellular contacts but was found at the cell periphery, in contrast to CaCo-2_WT_ cells, where tricellulin was stained only at tricellular junctions (Figure 7A, middle panel). Ectopic expression of LSR-EGFP in CaCo-2_ΔLSR_ cells reverted the localization of tricellulin at tricellular contacts (Figure 7B). The cell adhesion molecule E-cadherin was distributed equally along the cell-cell contacts of both wild-type and LSR-deficient CaCo-2 cells (Figure 7A, lower panel).

It can be concluded from these findings that LSR is required for proper targeting of tricellulin to tricellular contacts and for maintenance of a proper epithelial sheet geometry in CaCo-2 cells.

### LSR is required for the maintenance of the epithelial barrier function of CaCo-2 cells

**Figure 8:**
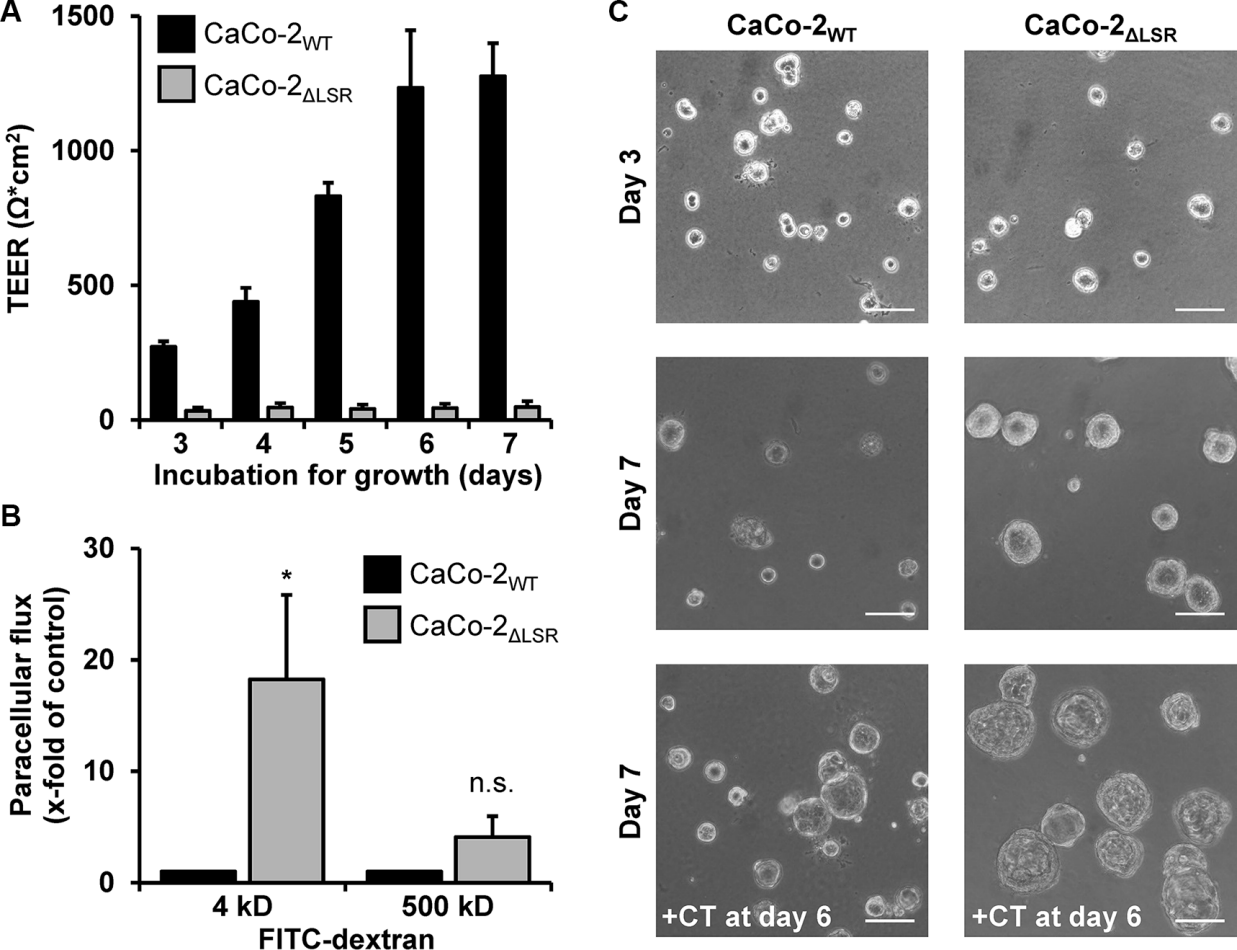
Assessing the epithelial barrier function of CaCo-2_WT_ and CaCo-2_ΔLSR_ cells (**A**) CaCo-2_WT_ and CaCo-2_ΔLSR_ cells were seeded into transwells and incubated for growth. Transepithelial electrical resistance (TEER) was measured after day 3 on a daily basis with a Volt-Ohm meter. TEER results are expressed as absolute TEER values (Ω*cm^2^) with black bars representing CaCo-2_WT_ and grey bars representing CaCo-2_ΔLSR_ cells. Bar graphs represent quantitative analysis of three independent experiments. The values represent mean ± SEM. (**B**) FITC-dextrans with the size of either 4 kDa or 500 kDa were added to the apical side of confluent CaCo-2_WT_ and CaCo-2_ΔLSR_ cell monolayer. Following an incubation period of 3 h, medium from the basolateral site of the cell monolayer was collected and analyzed for the presence of fluorescence. The paracellular flux of each FITC-dextran is shown as x-fold increase in CaCo-2_WT_ cells (black bars). Grey bars represent the results obtained with CaCo-2_ΔLSR_ cells. Bar graphs represent quantitative analysis of three independent experiments. The values represent mean ± SEM. *p < 0.05; n.s., not significant. (**C**) CaCo-2_WT_ and CaCo-2_ΔLSR_ cells were grown in Matrigel-coated wells and pictures of polarized cysts were taken at day 3 (top panel) and day 7 (middle panel). In parallel, both wild-type and LSR knockout cysts were treated with cholera toxin (100 ng/ml) for 24 h (+CT) prior to microscopy at day 7 (bottom panel). Scale bars represent 100 μM.

From the microscopic observations shown in Figure 6, we hypothesized that the epithelial barrier function of CaCo-2_ΔLSR_ cells might be affected by the loss of LSR. To test this hypothesis, we performed transepithelial electrical resistance (TEER) measurements with confluently growing CaCo-2_WT_ and CaCo-2_ΔLSR_ cells. We observed for CaCo-2_WT_ cells a time-dependent increase of the transepithelial electrical resistance that reached its maximum after approximately seven days (Figure 8A, black bars). In contrast, no increase of the transepithelial electrical resistance could be measured when CaCo-2_ΔLSR_ cells were grown for the same time period (Figure 8A, grey bars).

To substantiate that the CaCo-2_ΔLSR_ cell monolayer exhibits leakiness to small molecules, we performed a FITC (fluorescein isothiocyanate)-dextran permeability assay. CaCo-2_WT_ and CaCo-2_ΔLSR_ cells were plated to confluency in culture inserts and were left to form differentiated monolayers for seven days. Then, FITC-dextrans with the sizes of 4 or 500 kDa were added to the apical site of the cell monolayer. After 3 h of incubation, medium from the basolateral site of the cell monolayer was collected and analyzed for the presence of fluorescence. As shown in Figure 8B, paracellular permeability was highly increased in CaCo-2_ΔLSR_ cells for 4 kDa FITC-dextran molecules but not for the 500 kDa FITC-dextran molecules (Figure 8B, grey bars). In contrast, the CaCo-2_WT_ cell monolayer was permeable neither for the 4 kDa nor for the 500 kDa FITC-dextran molecules (Figure 8B, black bars). Thus, opening of tricellular contacts by the loss of LSR might contribute to an increased permeability of the epithelial barrier for small molecules.

To further investigate the role of LSR in epithelial barrier formation, we analyzed CaCo-2_WT_ and CaCo-2_ΔLSR_ cells in a three-dimensional Matrigel model, in which the cells form polarized cysts [19]. As shown in Figure 8C, cysts from CaCo-2_WT_ cells remained rather small after seven days of cultivation (Figure 8C, middle panel). Interestingly, cysts formed by CaCo-2_ΔLSR_ cells were larger in size than cysts from CaCo-2_WT_ cells (Figure 8C, middle panel), which might be due to the increased proliferative potential of CaCo-2_ΔLSR_ cells. The lumen of both, CaCo-2_WT_ and CaCo-2_ΔLSR_ cysts, could be enlarged by overnight treatment with cholera toxin (CTX) that causes water influx and consequently water accumulation by stimulating the adenylate cyclase and opening of cystic fibrosis transmembrane conductance regulator (CFTR) chloride channels [20] (Figure 8C, lower panel). The CTX-induced expansion of the lumen of CaCo-2_ΔLSR_ cysts indicates that bicellular tight junctions and other cell-cell contacts are not fully diminished in these cells, even though they do not establish a TEER when grown as cell monolayer.

To exclude that the loss-of-function effects of CaCo-2_ΔLSR_ cells are due to off-target effects of Cas9, we generated a second CaCo-2 LSR knockout clone (CaCo-2_ΔLSR-2_) with an alternative Cas9 targeting sequence for LSR. As shown in Supplementary Figure S2, CaCo-2_ΔLSR-2_ cells lack LSR expression (Supplementary Figure S2A), exhibit the same disordered phenotype upon growth to confluency as CaCo-2_ΔLSR_ cells (Supplementary Figure S2B), show also an increased cell viability (Supplementary Figure S2C) and strongly decreased TEER values (Supplementary Figure S2D) when compared to CaCo-2_WT_ cells.

## DISCUSSION

The lipolysis-stimulated lipoprotein receptor (LSR) is a membrane protein with diverse (patho)physiological functions in mammalian cells. LSR acts as a lipoprotein receptor in the liver [2], it contributes to the formation of tricellular tight junctions in mammary epithelial cells [5], it is required for the formation of the blood-brain barrier [9], and, finally, it acts as a cell entry receptor for clostridial iota-like toxins [7, 8].

Of increasing interest is the association of LSR with the progression and metastatic spread of various cancers. According to Yang and colleagues, LSR is one of the most up-regulated genes related to development of visible metastasis in a mouse mammary tumor model [10]. Another study found that LSR may be involved in both invasion and cellular movement in bladder cancer [11]. Furthermore, LSR was suggested to be a good tumor marker, because expression levels of LSR are often associated with a poor prognosis factor in human colon cancer [13]. A more recent study found a direct role of LSR in promoting aggressive breast cancer behavior [12]. A new report from Shimada *et al*. has shown that knockdown of LSR by siRNA in the endometrial cancer cell line Sawano, induced cell migration, invasion and proliferation [14].

These reports prompted us to investigate, if the loss of LSR affects tumor growth of colon carcinoma-derived cells. We chose the human colon carcinoma cell lines HCT116 and CaCo-2 for that purpose. LSR-deficient HCT116 cells were already available in our laboratory [15]. Here, we generated an LSR-deficient CaCo-2 cell line with CRISPR/Cas9, a gene editing technology that allows the targeted deletion of genes in mammalian cells [21]. To exclude off-target effects during knockout mutagenesis, we selected an LSR-specific gRNA from a genome-wide database of unique gRNAs computed and provided by the Church laboratory [21]. The knockout of LSR in CaCo-2 cells could be verified by immunoblotting and by DNA sequencing. In addition, binding and intoxication experiments with the *C. difficile* binary toxin CDT that uses LSR for cell entry, confirmed lack of LSR expression in CRISPR/Cas9-generated LSR knockout CaCo-2 cells.

At first, we studied the tumor-promoting role of LSR in a mouse xenograft model with wild-type and LSR-deficient HCT116 and CaCo-2 cells. Loss of LSR had no influence on growth of tumors formed by HCT116 cells. However, we noticed that tumors originating from CaCo-2 LSR knockout cells grew to a much smaller size than tumors from wild-type CaCo-2 cells. Interestingly, histological analysis of a CaCo-2 LSR knockout tumor revealed a less prominent stromal component, including few blood vessels, as well as increased cell death, including necrosis with karyorrhexis and karyolysis and also apoptosis. Thus, LSR deficiency might hamper tumor xenograft growth of CaCo-2 cells by leading to decreased angiogenesis and, consequently, to cell death induced by (oxygen) undersupply.

A study from Herbsleb and colleagues showed that RNAi-mediated knockdown of LSR in the bladder cancer cell line SW780 caused upregulation of gene networks implicated in cell growth and proliferation and thus to increased invasive cell behavior in a Matrigel-based invasion assay [11]. Very recently, it was reported also for the endometrial cancer cell line Sawano that LSR knockdown increased cell proliferation [14]. However, in Hs578t cells, increased cell proliferation was observed upon overexpression of LSR [12]. We found for CaCo-2 cells that the proliferative potential and metabolic turnover rate was increased under *in vitro* conditions upon knockout of LSR. This was not the case for HCT116 cells, where the knockout of LSR had no effect on their proliferative and metabolic potential.

Due to the observed differences in tumor growth and cell proliferation, we focused our attention on CaCo-2 wild-type and LSR knockout cells. The comparison of cell monolayer of wild-type and LSR deficient CaCo-2 cells uncovered that LSR-deficient CaCo-2 cells, in contrast to wild-type cells, were not able to form a regular, mosaic-like cell pattern upon reaching confluency. LSR-deficient CaCo-2 cells showed a strongly disordered cell monolayer and an aberrant epithelial sheet geometry. According to Masuda and colleagues, LSR knockdown in the mammary epithelial cell line EpH4 caused mislocalization of tricellulin apart from tricellular contacts [5]. Supportingly, a recent study reported for the endometrial cancer cell line Sawano that tricellulin relocalized from the tricellular region to the bicellular region at the membrane upon LSR knockdown [14]. We could confirm mislocalization of tricellulin upon knockout of LSR also in CaCo-2 cells. Whether the mislocalization of tricellulin in CaCo-2 LSR knockout cells is directly responsible for the altered epithelial sheet geometry needs to be addressed in the future. In a recent study from Reaves and colleagues, LSR was expressed in the Hs578t claudin-low breast cell line that typically does not express detectable levels of LSR. Interestingly, the authors observed that after expression of LSR, Hs578t cells changed their morphology and grew in distinct clusters. In addition, LSR expression in Hs578t cells resulted in the upregulation of genes associated with tight junctions formation and cell adhesion [12]. We speculate that the lack of LSR expression in CaCo-2 cells might suppress the mesenchymal-to-epithelial transition (MET) and promote a more mesenchymal-like growth phenotype in cell monolayers. Transitions between epithelial and mesenchymal states typically include changes in the expression levels of epithelial cadherin (E-cadherin) [22]. Interestingly, we did not observe any changes in cell surface-expression of E-cadherin between confluent monolayers of wild-type and LSR-deficient CaCo-2 cells.

The aberrant changes in cell morphology upon knockout of LSR in CaCo-2 cells prompted us to test whether these cells are able to form an intact, tight cell monolayer. By measuring the transepithelial electrical resistance (TEER) or the paracellular flux with fluorescent tracers, we could demonstrate that the loss of LSR in CaCo-2 cells renders the cells unable to form a tight cell monolayer. However, the size-selective permeability of the cell monolayer indicated that other cell-cell contacts, e.g. bicellular tight junctions, are not fully diminished in CaCo-2 LSR knockout cells [23]. This is supported by our results obtained with 3D cultures, where CaCo-2 wild-type cysts and the LSR knockout counterparts could both be further enlarged after incubation with cholera toxin. Other groups also observed strongly decreased TEER values in cells that either naturally lack LSR expression (Hs578t cells) or upon knockdown of LSR (EpH4 cells), respectively [5, 12]. Importantly, an increase of TEER was observed in Hs578t cells that ectopically expressed LSR [12]. In line with these observations, LSR seems to play an important role in the formation of a tight, impermeable epithelial barrier also in CaCo-2 cells.

The loss of the epithelial barrier function due to knockout of LSR might affect the LSR-deficient CaCo-2 cells´ ability to maintain a high tumor interstitial fluid pressure (TIFP), which is a characteristic of most human and experimental solid tumors [24]. Interestingly, Hofmann and colleagues have shown in a xenograft tumor model that a low TIFP reduces tumor cell proliferation. The authors suggested that the mechanical stretch of the tumor cortex induced by the TIFP is a positive modulator of tumor proliferation [25]. Therefore, it will be of future interest to study the role of LSR for the maintenance of high TIFP levels in tumors.

What could be the reasons that, in contrast to CaCo-2 cells, cell proliferation and tumor xenograft growth was not changed in HCT116 cells upon knockout of LSR? We assume that the role of LSR in cell-cell contacts is less important in HCT116 cells that, in contrast to CaCo-2 cells, typically do not differentiate into enterocytes upon growth to confluence. Therefore, loss of LSR in these cells may not induce epithelial-to-mesenchymal transitions that would increase cell proliferation as observed for CaCo-2 cells *in vitro*. HCT116 cells might already maintain a mesenchymal-like growth behaviour at any cell density even in the presence of LSR. LSR-deficient CaCo-2 tumors may not be able to efficiently attract blood vessels leading to cell death by undersupply with oxygen and nutrients. It can be speculated that HCT116 cells have adapted to (hypoxic) tumor environments by LSR-independent activation of angiogenesis-specific genes.

LSR exists in several isoforms generated by alternative splicing [4, 15]. LSR isoforms can be subdivided into two groups according to the presence or absence of a transmembrane segment present in the middle part of the protein [15]. The way the different isoforms contribute to the various (patho)physiological functions of LSR and to cancer progression remains to be studied.

Taken together, our study highlights the crucial role of LSR for maintaining the morphology and epithelial barrier of CaCo-2 cells and for the growth of CaCo-2 tumors in a mouse xenograft model. The *in vitro* data presented here and in previous studies from other groups indicates that LSR negatively regulates cell proliferation. However, at *in vivo* conditions, this effect of LSR might be less important. In contrast, our data from tumor xenografts in mice showed that other functions of LSR are crucial for tumor growth. How the role of LSR in tricellular junctions formation or even in lipoprotein uptake is connected to cancer progression still remains to be studied. Identification of potential LSR-dependent signaling pathways or interaction partners will be crucial for placing the (patho)physiological roles of LSR in cancer into a coherent picture.

Fagan-Solis and colleagues have recently shown in an *in vitro* model that LSR-overexpressing breast cancer cells were highly sensitive to *C. perfringens* iota toxin that uses LSR for cell entry. Thus, the authors speculated that iota-toxin has the potential to be an effective, targeted therapy for breast cancer [26]. LSR might represent a promising oncotarget for clostridial iota-like toxins also in colon cancers cells that show increased levels of LSR at the cell surface. LSR-overexpressing cancer cells, independent of the role of LSR in these cells, are ideal oncotargets because they would be already susceptible to low doses of clostridial iota-like toxins and allow a rather selective killing.

## MATERIALS AND METHODS

### Cultivation, transfection and 3D cultures of CaCo-2 cells

CaCo-2 (ATCC^®^ HTB-37™) and HCT116 (ATCC^®^ CCL247™) cells were grown in DMEM (12 mM L-glutamine) supplemented with 10% (vol/vol) FCS, 1% (vol/vol) non-essential amino acids, 1% (vol/vol) sodium pyruvate, and 1% (vol/vol) penicillin/streptomycin, and incubated at 37°C with 5% (vol/vol) CO_2_ under humidified conditions. Only cells that were tested negative for mycoplasma contamination (VENOR^®^GEM; Biochrom GmbH, Berlin, Germany) were used in experiments. Transfections were performed either with PEI (polyethylenimine) or with Lipofectamine^®^ 2000 transfection reagent (Thermo Fisher Scientific, Waltham, MA, USA) according to the manufacturer´s instructions.

Three-dimensional (3D) cultures of CaCo-2 cells were set up in 4-well Falcon™ Culture Slides (Corning, USA). Ice-cold Matrigel (60 μl) was transferred into each well and then allowed to solidify at 37°C. CaCo-2 cells were resuspended in medium supplemented with 2% Matrigel and plated at 5000 cells per well. Cysts formed during 6 days of cultivation at 37°C, while Matrigel-supplemented medium was replaced every 2–3 days.

### Generation of CaCo-2 LSR knockout cells via CRISPR/Cas9

CaCo-2 LSR knockout cells were generated via the CRISPR/Cas9 technology based on the protocol published by the Church laboratory [21]. Recently, we followed the same procedure to generate an LSR knockout in the HCT116 cell line [15]. Briefly, a guide RNA expression fragment was ordered as double-stranded DNA (gBlocks™; Integrated DNA Technologies, Leuven, Belgium) that consisted of an U6 promoter followed by a 20 bp protospacer, a PAM (protospacer adjacent motif) sequence, and a scaffold and terminator sequence. The protospacer sequence was complementary to a sequence in exon 2 of the LSR gene and reads as follows: 5′-GGACAGCGTGCGCACCGTCA-3′. The gBlock was inserted into the pCR-Blunt II-TOPO vector and then transfected into CaCo-2 cells together with the human codon-optimized Cas9 expression plasmid pcDNA3.3-TOPO/hCas9 (Addgene plasmid #41815). Transfected cells were selected with neomycin and then treated with CDT (4 nM CDTa and 5 nM CDTb) to select for toxin-resistant CaCo-2 LSR knockout cells. Finally, a single CaCo-2 LSR knockout clone was isolated via limiting dilution cloning and designated CaCo-2_ΔLSR_. A base pair insertion in exon 2 of the LSR gene of CaCo-2_ΔLSR_ cells, resulting in a frame-shift mutation, was confirmed by PCR and Sanger sequencing of the genomic region flanking the mutation site. A second CaCo-2 LSR knockout clone (CaCo-2_ΔLSR-2_) was generated essentially as described above, but with protospacer sequence 5′-GGCCGGAGGATTACCATCACCGG-3′ targeting also exon 2 of LSR but downstream of the protospacer sequence for generating CaCo-2_ΔLSR_. Targeting sequences of guide RNAs for CRISPR/Cas9-mediated DNA cleavage are illustrated in Supplementary Figure S1.

### Plasmids, proteins and toxins used in this study

The plasmid pEGFP-N1/LSR was used for the ectopic expression of LSR-EGFP in CaCo-2 cells and is described elsewhere [27].

CDTa and the precursor form of CDTb (pCDTb) were purified as recombinant proteins from the expression host *Bacillus megaterium* as described earlier [28]. CDTb was obtained by proteolytic activation of pCDTb [29, 30]. Intoxication experiments with CaCo-2 cells were performed by adding 4 nM CDTa together with 5 nM CDTb directly to the cell culture medium.

Recombinant production and DyLight488-labeling of the receptor-binding domain (RBD) are described elsewhere [27]. Cholera toxin from *Vibrio cholerae* was obtained from Sigma-Aldrich (C8052).

### Analysis of cell surface-binding of DyLight-labelled proteins by flow cytometry

Binding of DyLight488-labelled proteins to the cell surface of CaCo-2 cells was analyzed by flow cytometry. The cells were detached from culture plates with PBS, complemented with 10 mM EDTA, washed once with medium, and, eventually, kept on ice. Typically, 3 × 10^5^ cells in a total volume of 100 μl were incubated with 2 μM of DyLight-labelled protein for 10 min on ice. The cells were then washed twice with PBS and subjected to flow cytometric analysis, using the BD FACSCalibur platform. Cell surface-bound fluorescence was detected with an argon-ion laser (488 nm) and a 530-nm-bandpass filter (FITC). Flowing Software (version 2.5.0) was used for the computational analysis of the FACS measurements.

### Preparation of whole-cell lysates and detection of proteins by immunoblotting

Whole-cell lysates were prepared by washing cells directly in wells with ice-cold PBS, followed by lysis on ice in RIPA buffer (50 mM Tris-HCl/pH 7.5, 1% (wt/vol) Triton X-100, 1% (wt/vol) glycerol, 0.5% sodium deoxycholate, 0.1% sodium dodecylsulphate, 137 mM sodium chloride, 1 mM sodium orthovanadate, and 0.5 mM EDTA) complemented with Complete™ protease inhibitor cocktail. Cell debris was removed by centrifugation (21,000 g, 10 min, 4°C) and the protein concentration of the supernatant was measured with BCA protein assay kit (Uptima, Montluçon, France).

LSR was detected with a rabbit polyclonal anti-LSR antibody (clone X-25) (dilution 1:500; sc-133765; Santa Cruz) and tubulin with a mouse monoclonal anti-tubulin antibody (dilution 1:10000; T9026; Sigma-Aldrich). Horseradish peroxidase-conjugated donkey anti-mouse IgG (H&L) (#610–703–124; Rockland) or goat anti-rabbit IgG (H&L) (#7074; Cell Signaling) antibodies (dilutions 1:3000) were used as secondary antibodies. Antibody signals were developed by enhanced chemiluminescence reaction.

### Microscopy, immunostaining and cell surface staining

CDT-induced cell rounding of CaCo-2 cells was analyzed directly in wells by using an inverted light microscope (Axiovert 25, Carl Zeiss) with an inbuilt digital camera (AxioCam HRc). Confocal fluorescence microscopy was performed with an inverted microscope (Axiovert 200M; Carl Zeiss) equipped with Plan-Apochromat objectives, a spinning-disk head (Yokogawa) with emission filters, and solid-state laser lines (488 and 561 nm). Confocal fluorescence images were collected with a CoolSNAP-HQ2 digital camera (Roper Scientific) followed by processing with Metamorph imaging software (Universal Imaging). Immunostaining of LSR, tricellulin and E-cadherin in fixed cells were performed with rabbit anti-LSR (sc-133765; Santa Cruz), rabbit anti-tricellulin (700191; Thermo Fisher Scientific) and mouse anti-E-cadherin (610181; BD Biosciences) antibody, respectively. Alexa 488 goat anti-mouse IgG (A11001; Thermo Fisher Scientific), Alexa 488 goat anti-rabbit IgG (A11008; Thermo Fisher Scientific) and Alexa 568 goat anti-rabbit IgG (A11011; Thermo Fisher Scientific) were used as secondary antibodies. Rhodamine-labeled wheat germ agglutinin (WGA) (VC-RL-1022-M005; Axxora) was used to stain the cell surface of CaCo-2 cells. Cell nuclei were stained with DAPI.

### Human tumor xenografts in mice and histological analysis of xenograft tumors

All procedures were carried out in accordance with local animal ethics committees (G-12/34 and G-13/116). Subcutaneous xenografts were generated by injecting 2 × 10^6^ CaCo-2 or 5 × 10^5^ HCT116 cells (in 100 μl PBS) into the right flank of male, 8-week old, immunodeficient BALB/c Rag2^-/-^ mice. Prior to this, cells were transduced with firefly-luciferase to enable for *in vivo* bioluminescence imaging (BLI) of growing tumors [31]. *In vivo* BLI was performed as described earlier [32]. In addition to *in vivo* BLI, volumetric measurements of growing tumors were performed with the help of a caliper. Eventually, tumors were subjected to histologic processing. Formalin-fixed and paraffin-embedded xenograft tumors were cut (3 μm), deparaffinized and subjected to hematoxylin and eosin (H&E) staining according to standard techniques [33].

### Measurement of transepithelial electrical resistance and paracellular flux assay

For analysis of transepithelial electrical resistance (TEER) of Caco-2 cell monolayers, cells were seeded on transwells (Millicell; Millipore) and grown to confluency with daily medium exchanges. The increase of TEER was measured after day 3 on a daily basis by the use of a Volt-Ohm meter (World Precision Instruments, USA). Eventually, a paracellular flux assay was performed with the confluent CaCo-2 cell monolayer. 4 kDa and 500 kDa FITC-dextrans were used as fluorescent tracers to measure the paracellular permeability. Briefly, the FITC-dextrans were added directly to the medium on the apical site of the cell monolayer (donor compartment) with a final concentration of 2 mg/ml. After 3 h of incubation at 37°C, samples of 200 μl volume were removed from the basolateral site of the cell monolayer (acceptor compartment) and analyzed on a multiwell fluorescence reader (Infinite M200; Tecan; excitation: 493 nm, emission: 518 nm).

### Cell exclusion zone assay

In each of two chambers of a silicon-based culture insert (Ibidi, Martinsried, Germany), 4 × 10^4^ CaCo-2 or HCT116 cells were seeded and allowed to grow to confluency only in the designated areas. A cell-free gap of approximately 500 μm was created after removing the culture insert. Gap closure by proliferating and/or migrating cells was then observed for 48 h by time-lapse microscopy. The area of the cell-free gap was quantified with Axiovision SE64 software (Carl Zeiss, Jena, Germany).

### EdU proliferation assay

Cell proliferation was determined in CaCo-2 or HCT116 cells using a 5-ethynyl-2′-deoxyuridine (5-EdU) cell proliferation kit (EdU Click 2–488 ROTI^®^ kit; Carl Roth, Karlsruhe, Germany), which measures newly synthesized DNA. Briefly, in each well of a 96-well plate, 2.5 × 10^4^ cells were seeded. The next day, cells were incubated with 10 μM 5-EdU overnight, prior to fixation, permeabilization and detection of the incorporated 5-EdU by click chemistry with the fluorescent dye 6-FAM azide according to manufacturer´s instructions. Labelled cells were analyzed via fluorescence microscopy and subsequent quantification of total fluorescence intensity was performed with Metamorph software.

### Cell viability assay

The metabolic activity of CaCo-2 or HCT116 cells (cell viability) was determined with the CellTiter-Blue^®^ cell viability assay (Promega) following the manufacturer's protocol. Fluorescent products were measured on a multiwell fluorescence reader (Infinite M200; Tecan).

### Statistics

For statistical analysis of the data, experiments were repeated three times unless stated otherwise and data are presented as mean ± SEM. Student´s *t*-test was used for pairwise comparison of the data and significance calculations. *P* values < 0.05 were considered statistically significant.

## SUPPLEMENTARY MATERIALS FIGURES


