# Supplementary material for: Loss of LSR affects epithelial barrier integrity and tumor xenograft growth of CaCo-2 cells
Source: Oncotarget. 2016 Jul 6;8(23):37009–22. doi: 10.18632/oncotarget.10425 (PMC5514888; doi:10.18632/oncotarget.10425)
Supplement: Supplementary file 1 [file oncotarget-08-37009-s001.pdf]

Loss of LSR affects epithelial barrier integrity and tumor xenograft growth of CaCo-2 cells

Supplementary Materials

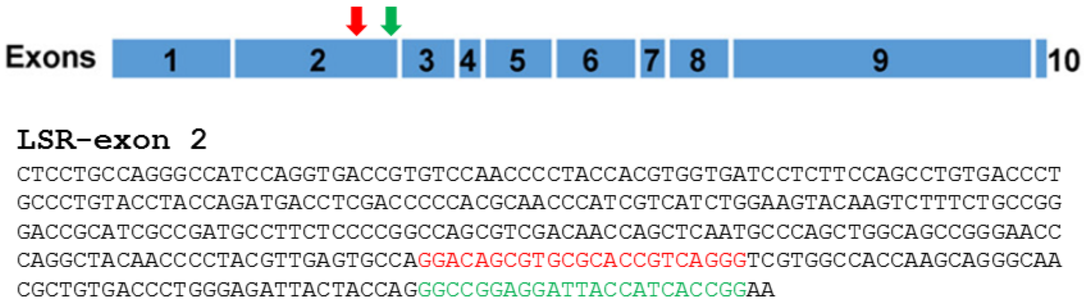

Supplementary Figure S1: Targeting sequences of guide RNAs for CRISPR/Cas9-mediated DNA cleavage. Gene architecture of human LSR gene (exons 1 to 10) is shown (upper panel) and DNA sequence of LSR exon 2 with highlighted sequences indicating targets of the guide RNAs for Cas9 (lower panel). Red, Cas9 targeting sequence for generating CaCo-$2_{\Delta LSR}$. Green, Cas9 targeting sequence for generating CaCo-$2_{\Delta LSR\text{-}2}$.

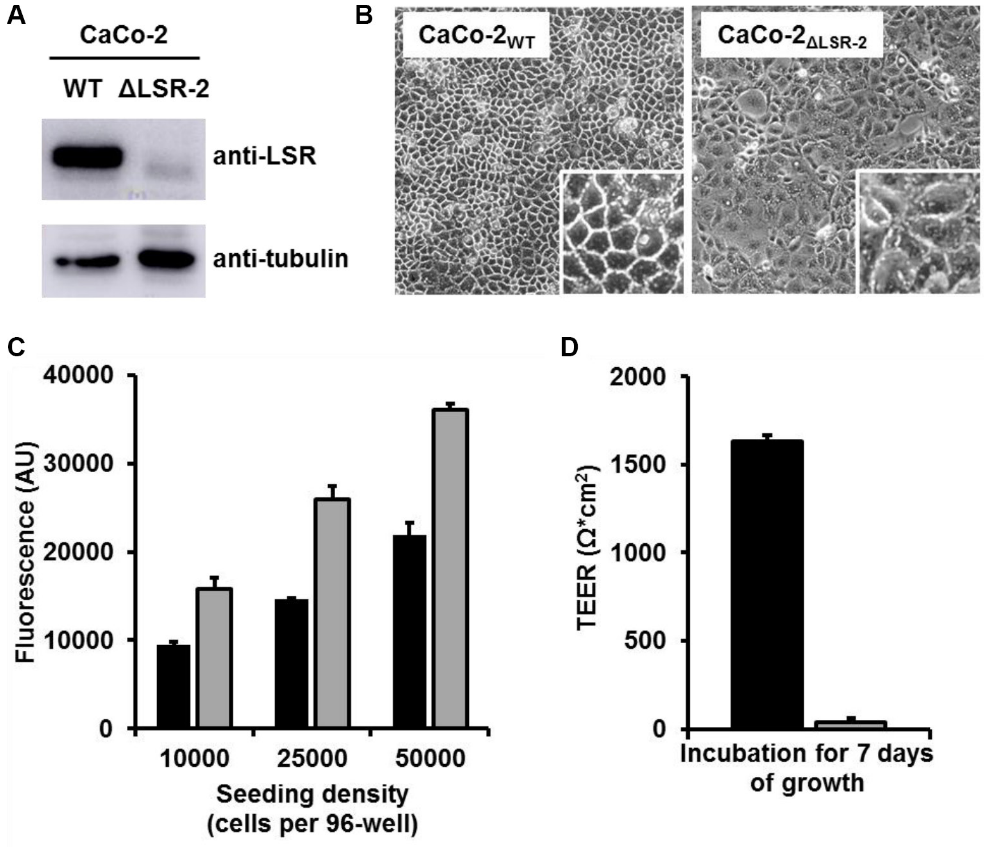

Supplementary Figure S2: Evaluation of CaCo-2$_{\Delta LSR-2}$ cells. (**A**) LSR immunoblot with whole-cell lysates from CaCo-2$_{WT}$ (WT) and CaCo-2$_{\Delta LSR-2}$ (ΔLSR-2) cells (top panel). Equal loading of samples was verified by detecting tubulin with a specific antibody (bottom panel). (**B**) Transmitted light microscopy images of confluent cell monolayers from CaCo-2$_{WT}$ and CaCo-2$_{\Delta LSR-2}$ cells. (**C**) Cell viability assay. Absolute resorufin fluorescence values are presented in arbitrary units (AU). Black bars represent fluorescence values obtained with CaCo-2$_{WT}$ and grey bars with CaCo-2$_{\Delta LSR-2}$ cells, respectively. Bar graphs represent quantitative analysis of triplicates. The values represent mean ±SEM. (**D**) CaCo-2$_{WT}$ and CaCo-2$_{\Delta LSR-2}$ cells were seeded into transwells and incubated for growth. Transepithelial electrical resistance (TEER) was measured after day 7 with a Volt-Ohm meter. TEER results are expressed as absolute TEER values (Ω*cm^2) with black bars representing CaCo-2$_{WT}$ and grey bars representing CaCo-2$_{\Delta LSR-2}$ cells. Bar graphs represent quantitative analysis of triplicates. The values represent mean ± SEM.
